# Effect of center of rotation of angulation‐based leveling osteotomy on ex vivo stifle joint stability following cranial cruciate ligament transection and medial meniscal release with and without a hamstring load

**DOI:** 10.1111/vsu.13801

**Published:** 2022-03-15

**Authors:** Parisa Mazdarani, Mir Sepehr Pedram, James E. Miles

**Affiliations:** ^1^ Department of Veterinary Clinical Sciences University of Copenhagen Frederiksberg Denmark; ^2^ Department of Surgery and Radiology, Faculty of Veterinary Medicine University of Tehran Tehran Iran

## Abstract

**Objective:**

To evaluate the effect of center of rotation of angulation (CORA)‐based leveling osteotomy (CBLO) and hamstring load on stifle stability following cranial cruciate ligament transection (CCLx) and medial meniscal release (MMR).

**Study design:**

Ex vivo experimental study.

**Sample population:**

Cadaver hind limb preparations (*n* = 7).

**Methods:**

After instrumentation, constant quadriceps and gastrocnemius loads with an optional hamstring load in a 3:1:0.6 ratio were applied, and stifles were extended from fully flexed using an electrical motor during fluoroscopic recording. The recording process was repeated after each of CCLx, MMR and CBLO and the extracted landmark coordinates were used for calculation of cranial tibial translation (CTT) and patellar ligament angle (PTA).

**Results:**

Mean initial tibial plateau angle was 28.1°: post‐CBLO the mean was 9.7°. Cranial tibial translation developed from 50° and 75° with CCLx and MMR respectively (*p* < .04, < .02) without hamstring loading. Hamstring loading mitigated CTT due to CCLx and delayed CTT until 120° for MMR (*P* < .02) in this model. CBLO prevented CTT, except at 140° without hamstring loading (*P* = .01). Similar results were seen for PTA, but CBLO curves were parallel to and lower than intact values at all tested angles (*P* < .04), consistent with induced effective joint flexion.

**Conclusion:**

CBLO to a target tibial plateau angle of 10° largely eliminated CTT induced by CCLx and MMR. Hamstring loads of 20% quadriceps load improved stifle stability in this model.

**Impact:**

Stifle stability following CBLO appears to be multifactorial and depends on meniscal integrity, joint angle, and hamstring strength.

## 1 INTRODUCTION

The cranial cruciate ligament (CCL) is the main limiter of cranial tibial translation relative to the femur and of hyperextension of the stifle joint in the dog.^1^ A CCL deficiency leads to altered kinematics and stresses in the femorotibial cartilage, which induce rapid progression of osteoarthritis and predispose to secondary meniscal injury.^2^, ^3^, ^4^ Cranial cruciate ligament rupture and medial meniscal injuries often occur in combination especially in larger dogs, and with longstanding stifle instability and cranial tibial translation.^5^

Surgical procedures for treating the CCL‐deficient stifle aim to eliminate cranial tibial translation (CTT) during weight bearing.^6^ The center of rotation of angulation (CORA)‐based leveling osteotomy (CBLO) is a recently described technique used to eliminate CTT in CCL‐deficient stifles, which manages the tibial plateau slope as if it were an angular deformity.^7^ If the center of a radial osteotomy (angulation correction axis or ACA) is positioned at the CORA, the proximal tibial epiphysis becomes aligned with the tibial diaphysis,^8^ and as a result the tibial plateau angle (TPA) and anatomical‐mechanical axis angle are reduced.^7^, ^9^, ^10^ Placement of the ACA away from the CORA can result in secondary translation and increased or decreased TPA following rotation of the proximal fragment.^8^, ^10^ In comparison with tibial plateau leveling osteotomy (TPLO), CBLO may reduce the risk of articular cartilage lesions in dogs with complete CCL rupture.^11^ Apart from one in silico study comparing stability with TPLO and CBLO,^12^ the effectiveness of CBLO in stabilizing the cruciate‐deficient stifle, most specifically the lack of caudal thrust and reduction but not elimination of cranial thrust,^11^, ^13^ has not been demonstrated biomechanically in either in vivo or ex vivo studies.

Theoretical modeling of the canine stifle has demonstrated that reducing the TPA alone does not completely neutralize the forces that lead to cranial tibial translation.^14^ This has been demonstrated in vivo by fluoroscopic evaluation of walking dogs post‐TPLO, which demonstrated reduced CTT, although caudal translation was observed in 10/16 dogs and persistent CTT in 5/16, despite all post‐TPLO TPA values lying within a typically accepted range.^15^ Stifle stabilization in dogs may rely on muscular support because of their steeper tibial slope in comparison with humans:^16^ the quadriceps, gastrocnemius, and biceps femoris muscles in combination appear important in maintaining joint stability.^16^, ^17^ Both quadriceps contraction and gastrocnemius contraction can generate cranial tibial translation,^18^, ^19^ whereas the flexors of the stifle joint (hamstring muscles) actively oppose this movement.^17^, ^20^ Abnormal hamstring activity has been documented in both hindlimbs of dogs unilaterally affected by CCL rupture,^21^ and hamstring exercises are considered important in postoperative physiotherapy,^22^ but ex vivo evidence for the effectiveness of hamstring loads is lacking in dogs.

Our aims were to (1) determine the effects of CBLO on the ex vivo stability of the stifle joints across a wide range of stifle angles following CCL transection (CCLx) and medial meniscal release (MMR) to create a maximally unstable joint situation, and (2) determine whether a simulated hamstring load is effective in maintaining the stability of the stifle joint following CCLx, MMR, and CBLO. We hypothesized that CBLO would eliminate CTT across a wide range of stifle flexion angles, and that the hamstring load would improve stifle stability.

## 2 MATERIALS AND METHODS

### 2.1 Limb preparation

**FIGURE 1:**
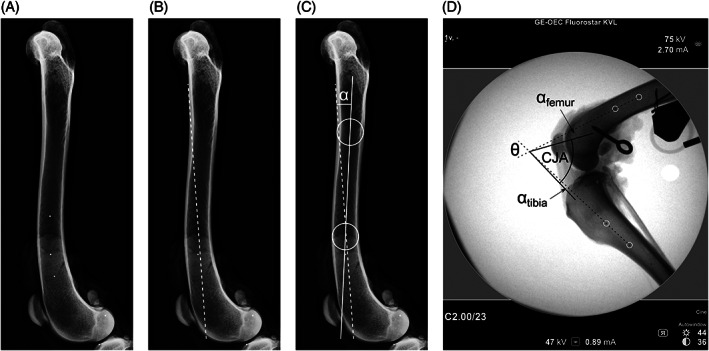
Diaphyseal marker beads and axis conversion. Example femur with implanted beads in the distal diaphysis (A). Two beads can be used to define a unique axis for this femur (dotted line, B). This axis can be converted to the standard axis (solid line, C) used for this study, which is drawn between diaphyseal centers at one‐third and 2‐thirds of the femoral length by use of a correction angle (alpha). A similar approach was used for the tibia. Still image taken from one fluoroscopic recording (D): Due to the limited extent of the fluoroscopic image, which excluded the proximal and distal halves of the femur and tibia, respectively, use of marker beads was necessary for joint angle derivation. Marker axes (dashed) have been extended from the middle and distal beads (femur) and proximal and middle beads (tibia) highlighted by white circles. Correction angles of −10.3° and +3.3° between these bead pairs and the bone axes were calculated for this femur and tibia, respectively. The caudal joint angle was then calculated as $\mathrm{CJA}=\theta +\alpha_{\text{femur}}+\alpha_{\text{tibia}}$

Seven left hind limbs, without evidence of stifle pathology on palpation or radiography, were harvested from client‐owned canine cadavers euthanized for reasons unrelated to this study and prepared as previously described.^23^ Written permission was obtained from the owners and approval was given by the ethical committee at the Department of Veterinary Clinical Sciences, University of Copenhagen. Body mass and the distance between the medial malleolus and the center of the common calcaneal tendon insertion (for later attachment of the gastrocnemius load) were measured before harvesting each limb. Removal of muscles and soft tissues not related to the stifle joint was performed, as well as hip and talocrural disarticulation. Prepared limbs were covered with saline‐soaked swabs, and stored in plastic bags at −22°C. Before use they were thawed at 5 °C for 24 h. Through 2 separate craniomedial and caudal arthrotomy approaches, 1 mm diameter stainless steel beads (Frits Pedersen; Copenhagen, Denmark) were placed at the centers of the origin and insertion of the CCL. Three additional beads were implanted along each distal femoral and proximal tibial diaphysis as axis markers. These were necessary as the fluoroscopic image did not include the proximal femur or distal tibia. The craniomedial arthrotomy was sutured with 3.5 metric polyglactin 910 and appropriate positioning of the beads in the origin and insertion of the CCL was confirmed radiographically. Full lateral radiographs of the femur and tibia were used to calculate correction angles between the diaphyseal bead locations and the anatomical axes of these bones (Figure 1).

### 2.2 Limb instrumentation

Instrumentation was as previously described.^23^ To mimic the insertion of the gastrocnemius muscle, a 10‐hole 3.5 mm dynamic compression plate (DCP), bent at 90°, was fixed to the craniodistal surface of the tibia with 2 screws, with the bent end directed caudally. A pulley wheel was fixed to the caudodistal femoral diaphysis, at the origin of the gastrocnemius, using a threaded bolt and cerclage wire, and augmented with a screw eye hook for simulation of the gastrocnemius load. For simulating the loads of the quadriceps and hamstring muscles, bone tunnels were drilled craniocaudally in the proximal patella and mediolaterally in the proximal tibial metaphysis just distal to the joint surface and caudal to the medial collateral ligament. Two screw eye hooks were inserted into the caudolateral and caudomedial aspects of the proximal femoral metaphysis to direct the passage of the hamstring loading line. Two 4.0/4.8 mm positive‐profile external fixation pins were placed caudocranially in the femoral diaphysis for later mounting the limb construct. Mediolateral radiographs, with superimposition of the femoral condyles and a 90° caudal joint angle, were obtained with the beam centered over the stifle joint.

### 2.3 Construct mounting

**FIGURE 2:**
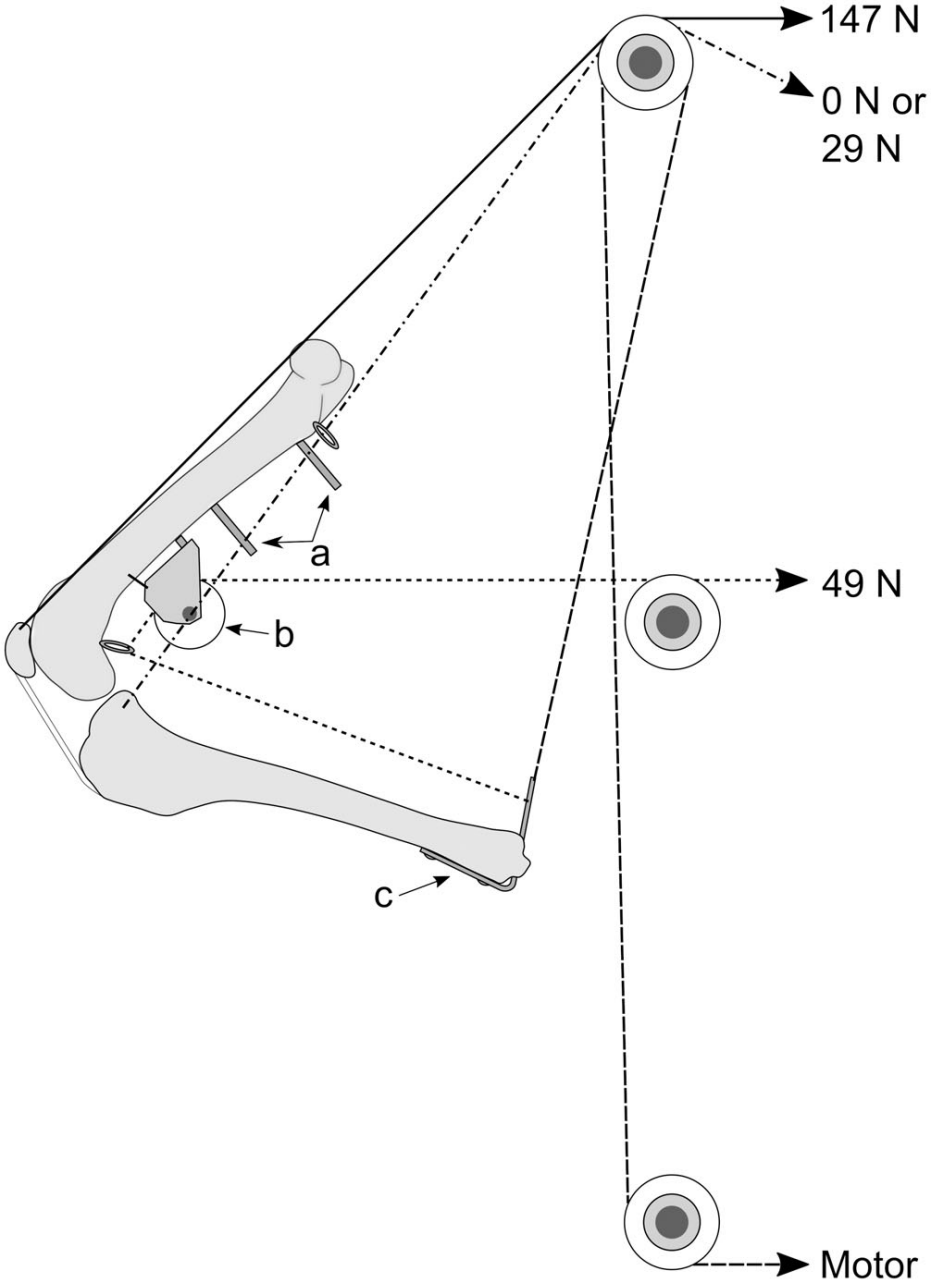
Experimental setup. Limbs were instrumented with positive‐profile external fixator pins (A) for attachment to the polycarbonate frame. A pulley wheel attached to the femur with a bolt and cerclage, and augmented with a screw eye hook, was used to simulate the gastrocnemius muscle line of action (B). A bent dynamic compression plate (C) attached to the distal tibia was used to mimic the calcaneus for attachment of the gastrocnemius load (49 N), and as a fixation point for a line attached to a geared motor to control joint extension. The quadriceps load (147 N) was attached to a tunnel in the proximal patella. An optional hamstring load (0 or 29 N) was attached to a tunnel through the proximocaudal tibial metaphysis, passing through screw eye hooks on either side of the proximal femoral metaphysis to avoid interference with other load lines or fixation implants. Loading lines were passed via additional pulleys to mimic physiological lines of action as far as possible. An additional counterweight (49 N, not shown) was attached to the distal tibia was used to assist extension

An external fixator frame attached to a 10 mm thick polycarbonate sheet was used for mounting the limb, as previously described.^23^ A 147 N weight simulating the quadriceps muscle was attached to the patellar tunnel using 0.75 mm diameter ultrahigh molecular weight polyethylene fishing line (Spectra 91 kg breaking strain line; Honeywell, Colonial Heights, Virginia). A similar line, loaded with 49 N, passing via the pulley wheel and distal screw‐eye hook was attached to the DCP at an equivalent distance of insertion of the common calcaneal tendon from the medial malleolus. A looped loading line was attached to a 29 N weight after passing through the proximal tibial metaphyseal bone tunnel and proximal screw eye hooks. Two additional lines, connected to the geared battery‐driven motor (Technics 870 4.5 V motor; LEGO, Billund, Denmark) and a 49 N counterweight were attached to the DCP to control stifle joint extension. All of the lines were placed in the physiological path with the aid of pulley wheels (Figure 2). A digital fluoroscopy unit (OEC Fluorostar 7900; GE Healthcare, Chicago, Illinois) was positioned with the recording head 10 cm (±0.5 cm) from, and parallel to, the polycarbonate sheet and centered over the stifle joint, ensuring maximum superimposition of the femoral condyles.

### 2.4 Test protocol

Starting from a flexion angle under 50°, the joint was gradually extended to full physiological extension using the electric motor at a rate of approximately 3° per second. The extension movement of the intact joint was recorded fluoroscopically at 25 frames per second within 2 × 30 s recording periods.

The CCL was transected through the reopened craniomedial arthrotomy. The above recording process, performed both with and without a hamstring load, was repeated after confirmation of CCL transection by the cranial drawer test, and closure of the arthrotomy.

The midbody of the medial meniscus was released using a number 11 scalpel blade directed 30° caudomedial‐craniolateral through a 1 cm approach caudal to the medial collateral ligament. This approach was not sutured to avoid inadvertent entrapment of the caudal horn. The 2‐step recording process was repeated after confirming the free movement of the caudal pole of the medial meniscus by probing with an instrument and observation to ensure complete transection.

The CBLO procedure was planned as previously described^7^ with a standard target TPA of 10° for all limbs, in order to identify the CORA location and the required correction angle. Briefly, the proximal tibial anatomical axis was defined offset 10° caudally to the perpendicular to the tibial plateau. The distal tibial anatomical axis was defined by the centers of the diaphysis at 50% of the tibial length and just proximal to the distal flare of the tibia.^24^ The intersection of these axes defined the location of the CORA, and the acute angle between them the correction angle. A radial osteotomy was performed centered over the CORA or as close distally to it as possible allowing placement of the head of a 3.5 mm polyaxial locking TPLO plate (N2 [UK] Ltd, Portsmouth, United Kingdom) in the proximal fragment. Additional stabilization was provided with pinning and tension band wiring rather than a headless compression screw for economy. Radiographs were obtained post‐CBLO for measurement of TPA, but no further adjustments were made based on these. The limb was mounted again and the final 2‐step recording process was accomplished.

### 2.5 Data extraction

The “scene filter” in VLC media player (VLC media player, Paris, France, www.videolan.org) was used to automatically extract still images from the video files at 2 s (50 frame) intervals. The stills of each individual run were named consecutively.

Predetermined landmarks were identified on each sequentially extracted image in freely available software (Image J; National Institutes of Health, Bethesda, Maryland, www.imagej.net) to obtain their coordinates. Joint angle (caudal angle between femoral and tibial anatomical axes), patellar ligament (tendon) angle (PTA) using the tibial plateau and cranial border of the patellar ligament, and CCL marker separation distance were calculated from these coordinates in a spreadsheet program (Excel; Microsoft, Redmond, Washington). The joint angle was calculated initially from the angle between marker bead pairs in the femur and tibia, and converted to the caudal joint angle by subtraction of the relevant correction angles for each bone (Figure 1D). Following CBLO, a combination of distal marker bead and pin‐bone interface was used for this correction because the proximal beads were typically obscured by the plate. CCL marker separation distance was used as a proxy for CTT as previously described.^23^, ^25^

Extracted data were interpolated with monotonic cubic splines (SRS1 splines for Excel; SRS1 Software, Boston, Maryland) at set joint angles from 40° to 145° with 5° intervals.

Measurements of Blumensaat's line (the roof of the intercondylar fossa)^26^ from still images of each recording were used to normalize all values between test runs (to correct for minor variations in fluoroscopy unit positioning) and between stifles (to correct for different stifle sizes), using the first recording of the first limb as the length standard.

### 2.6 Statistical analysis

Relationships between joint angle and both CCL marker separation distance and PTA were explored graphically. Statistical testing was performed using a commercial software package (SPSS v.27, IBM Corp., Armonk, New York). Data normality was assessed using the Shapiro‐Wilk test. Friedman's ANOVA was used for comparison of test runs for each group (with and without hamstring load) at each joint angle for CCL marker separation distance and PTA. Predetermined post hoc pairwise comparisons were made between intact versus CCLx, intact versus MMR, intact versus CBLO, and CBLO versus MMR. Test situations with and without hamstring loads were compared using Wilcoxon's signed rank test for CCLx, MMR, and CBLO. Associated *P* values were corrected using Holm's sequential Bonferroni correction for multiple comparisons: the significance level was set at .05.

Landmark identification repeatability and reproducibility were assessed using the within‐subject SD (wsSD) and type 2 intraclass correlation coefficient for absolute agreement (ICC _(1,2)_) by comparing spline‐derived values for marker separation and PTA for 2 observers and 5 readings of sequential still images from 1 stifle. Repeatability data were tested for homoscedasticity using Koenker’s test.

## 3 RESULTS

Mean body mass for these cadavers was 31.7 kg (SD 2.4 kg). The mean initial TPA was 28.1° (SD 4.3°), with a mean calculated correction angle of 20.7° (SD 4.5°) and mean final TPA following CBLO of 9.7° (SD 2.3°). No complications such as construct failure or fracture occurred during testing. Baseline CCL marker separation distance (100%) was 14.3 mm (SD 1.8 mm). Joint angles under fluoroscopic recording ranged from 43.7° (SD 6.1°) to 146.1° (SD 8.9°), and were censored to 50° to 140° after spline calculation to maximize sample size and statistical power, due to incomplete data at the limits of flexion and extension for some samples.

Data were confirmed as non‐normal (Shapiro‐Wilk test, *P* < .05). Homoscedasticity was confirmed with Koenker's test (*P* > .05). Intraobserver and interobserver repeatability and reproducibility for CCL marker separation distance and PTA showed good agreement based on wsSD values, but ICC values suggested that agreement was potentially poorer for CCL marker separation distance than for PTA (Table 1).

**TABLE 1:** Intraobserver repeatability and interobserver reproducibility for spline‐derived variables for 2 observers and 5 readings

	CCL marker separation distance		PTA	
	wsSD (%)	ICC_(1,2)_	wsSD (°)	ICC_(1,2)_
Observer 1	1.8 (1.6;2.1)	0.39 (0.16;0.63)	1.3 (1.1;1.4)	0.99 (0.98;0.99)
Observer 2	1.7 (1.5;2)	0.4 (0.15;0.65)	1.7 (1.5;2)	0.97 (0.91;0.99)
Interobserver	2.3 (1.5;3.2)	0.83 (0.48;0.94)	2.2 (1.4;3)	0.98 (0.95;0.99)

*Note*: Point estimates with 95% confidence intervals in parentheses.

Abbreviations: CCL, cranial cruciate ligament; ICC_(1,2)_, type 2 intraclass correlation coefficients for absolute agreement; PTA, patellar tendon (ligament) angle; wsSD, within‐subject SD.

### 3.1 Change in CCL marker separation distance with stifle joint extension

**FIGURE 3:**
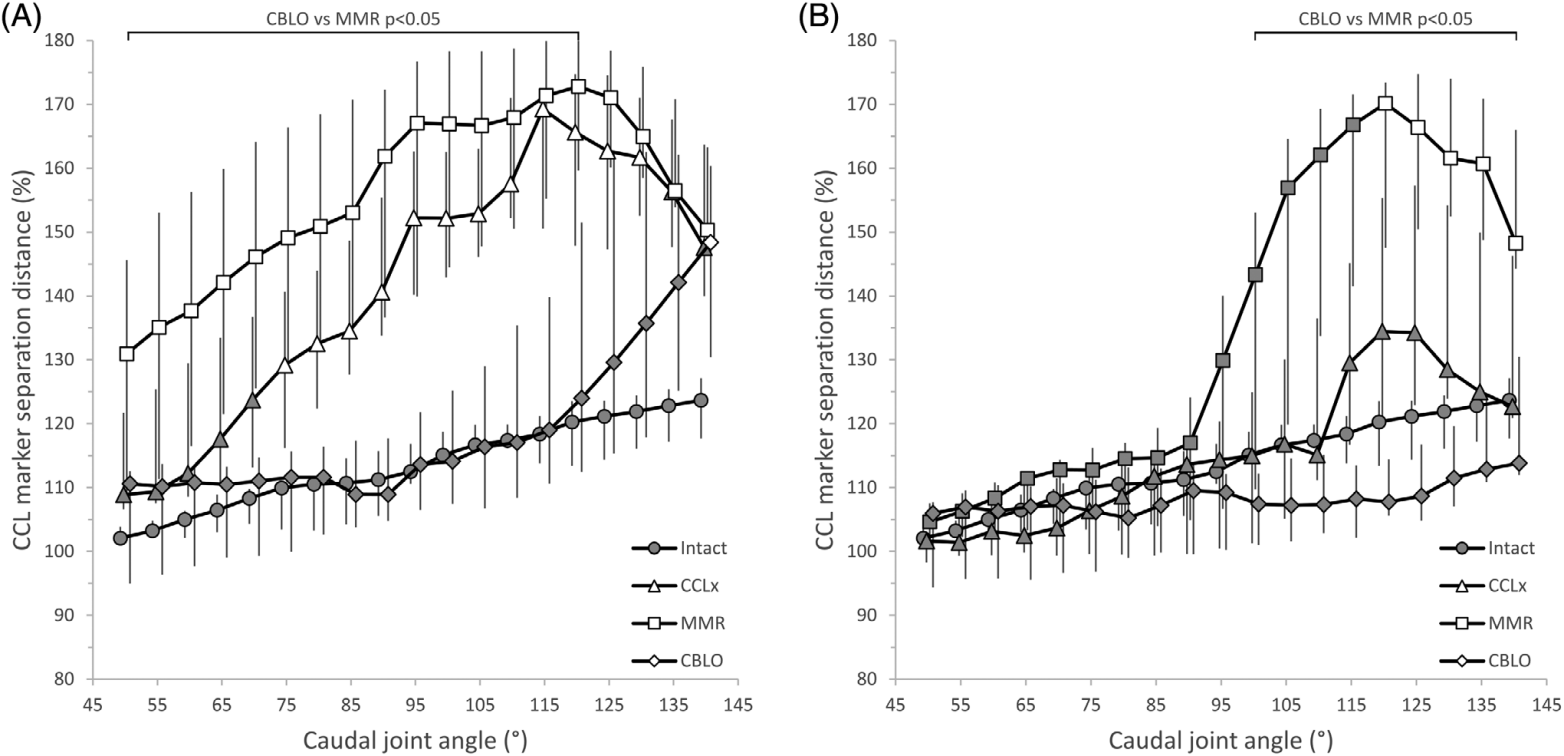
Cranial cruciate ligament marker separation distances. Median and interquartile range data are shown for intact, cranial cruciate ligament transected (CCLx), medial meniscal release (MMR) and CORA‐based leveling osteotomy (CBLO) stifles without (A) and with (B) a 29 N hamstring load. The caudal joint angle values have been staggered to enhance visualization of the error bars. Separation distances have been normalized to the intact situation for each stifle joint and between stifle joints using the length of Blumensaat's line, such that a value of 100% represents the initial separation. Shaded markers are not significantly different (*P* > .05) from the intact values (also shaded)

**FIGURE 4:**
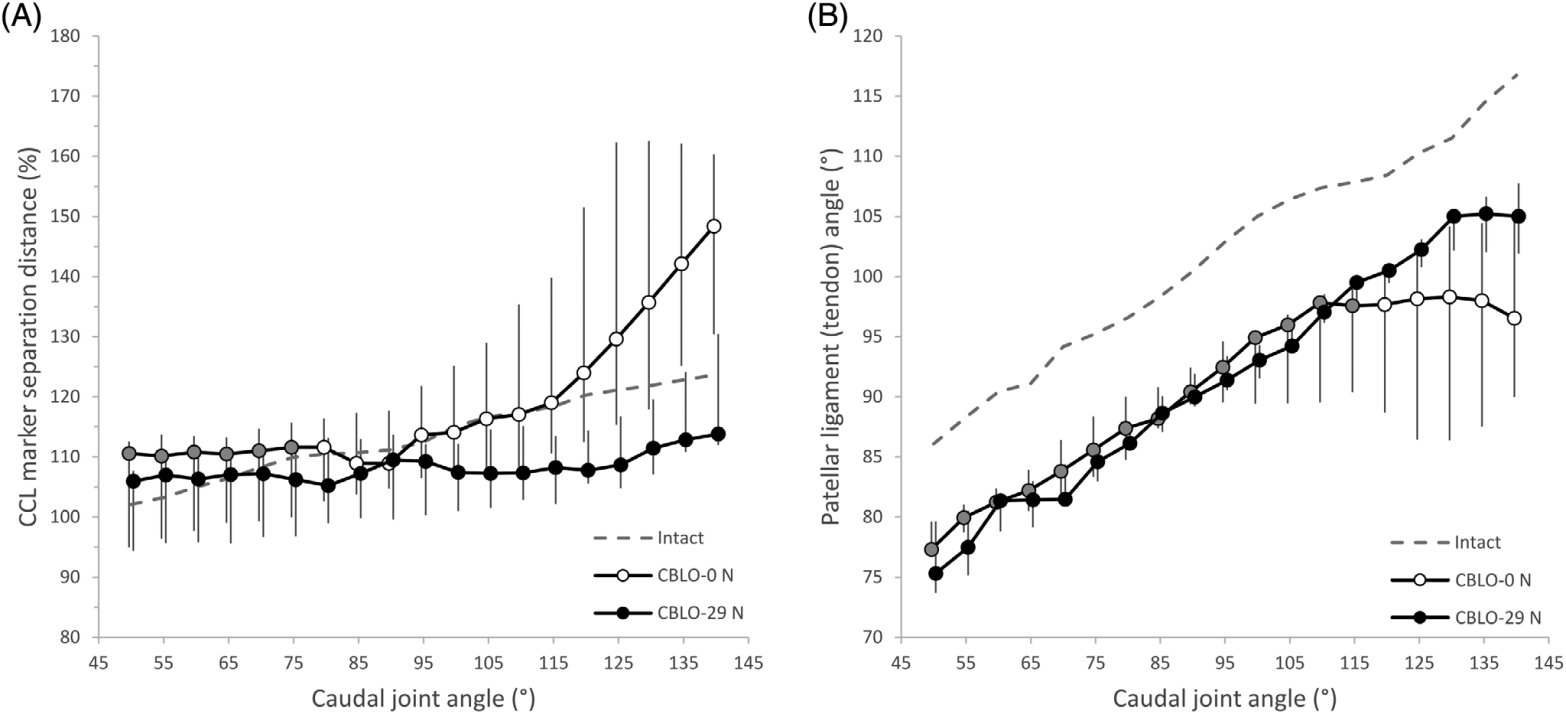
Effect of hamstring load on cranial cruciate ligament marker separation distances and patellar tendon angles for CORA‐based leveling osteotomy stifles. Median and interquartile range data are shown for cranial cruciate ligament separation distance (A) and patellar ligament angle (B) for stifles either without (CBLO‐0 N) or with (CBLO‐29 N) a 29 N simulated hamstring load across caudal joint angles of 50° to 140°. For comparison purposes, the intact situation is shown as a dashed line. Separation distances have been normalized to the intact situation for each stifle joint and between stifle joints using the length of Blumensaat's line, such that a value of 100% represents the initial separation. The caudal joint angle values have been staggered to enhance visualization of the error bars. Shaded CBLO‐0 N markers are not significantly different (*P* > .05) from the CBLO‐29 N values

Spline‐based marker separation distance gradually increased with increasing caudal joint angle in intact stifles (Figure 3A,B). Joints exhibited subluxation from 75° after CCLx (*P* < .04), which was more severe and began at 50° (*P* < .02) following MMR (Table S1). In the presence of the hamstring load, no difference in CCL marker separation was noted for CCLx, and joint subluxation occurred first from 120° with MMR (*P* < .02) (Table S2). Cranial tibial translation occurred in CBLO curves from 140° without the hamstring load (*P* = .01), but did not occur with the hamstring load. The CCLx and MMR curves showed less subluxation with the hamstring load from 50° to 135° (*P* = .018) and 50° to 140° (*P* < .04), respectively, whereas the CBLO curves only differed from 80° to 140° (*P* < .04) due to caudal tibial translation under the hamstring load (Figure 4A) (Table S3).

### 3.2 Change in PTA with stifle joint extension

**FIGURE 5:**
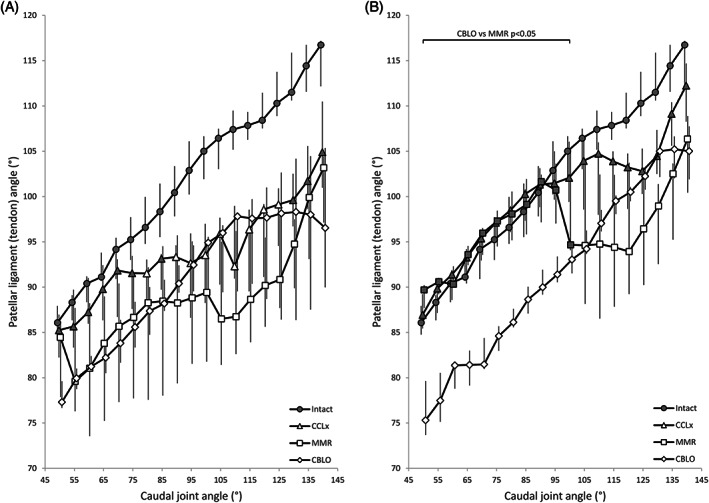
Patellar ligament (tendon) angles. Median and interquartile range data are shown for intact, cranial cruciate ligament transected (CCLx), medial meniscal release (MMR) and CORA‐based leveling osteotomy (CBLO) stifles without (A) and with (B) a 29 N hamstring load. The caudal joint angle values have been staggered to enhance visualization of the error bars. Shaded markers are not significantly different (*P* > .05) from the intact values (also shaded)

The spline‐based PTA increased almost linearly with increasing joint extension in intact stifles (Figure 5A,B). In the absence of the hamstring load, CCLx caused a reduction (*P* < .04) in PTA increase between 90° and 125°, while MMR caused a reduction (*P* < .02) from 50° to 140° (Table S4). Adding the hamstring load resulted in CCLx not producing a difference to the intact situation, while MMR only diverged from 105° to 140° (Table S5). CBLO resulted in a parallel and lower (*P* < .03) PTA curve in comparison with the intact situation, consistent with CBLO effectively placing the joint in a more flexed angle; the hamstring load yielded a similar curve below the intact curve (*P* < .04). The CCLx curve without the hamstring load was lower than with the hamstring load at all tested angles except 60°, 70°, and 140° (*P* < .04). For MMR, this held true at all angles except 125° to 135° (*P* < .04) (Table 6). The CBLO curve with the hamstring load was higher than that without from 120° to 140° (*P* < .04) (Figure 4B).

Based on an analysis of the CCL marker separation distance data, at a caudal joint angle of 125°, 3/7 stifles were stable (no CTT) without a hamstring load, and 5/7 were stable with a hamstring load.

## 4 DISCUSSION

In this ex vivo model, CBLO successfully eliminated joint subluxation following both CCLx and MMR across a wide range of joint angles. The hamstring load improved stifle stability in the flexed joint following MMR, but produced reductions in subluxation after CCLx alone throughout the joint angle range and beyond that expected from in vivo studies.^27^ In agreement with findings from earlier studies,^6^, ^23^, ^28^ we confirmed that the canine medial meniscus is a secondary restraint of CTT.

The muscle loads using in this study were taken from a previous model,^23^ with the quadriceps:gastrocnemius ratio lying within the interval for these muscles found in a quasi‐static model at 30%‐40% of stance,^29^ in the absence of more appropriate data. Higher gastrocnemius loads resulted in subluxation even in full joint flexion, indicating a relatively severe loading situation for testing joint stability.^23^ Most prior ex vivo models^28^, ^30^, ^31^, ^32^, ^33^, ^34^ have used simulated load bearing with indirect generation of muscle forces by direct linkages across the joint, but they did not report the magnitudes or ratio of these forces, which could vary with joint angle and subluxation. How closely these induced loads represent physiological loading ratios is unknown. Hamstring muscle loading was initially derived from published data for the semimembranosus and semitendinosus muscles.^29^ The published ratio of quadriceps to combined semimembranosus and semitendinosus forces of between 2.7:1 and 2:1 at 30%‐40% of stance, or 1.35:1 and 1:1 (assuming an equal contribution from the caudal biceps femoris) completely prevented subluxation in our pilot studies, and the balanced load was subsequently reduced to 29 N. Despite this apparently subphysiological load, the hamstring effect was more pronounced than expected, suggesting an overlooked factor in this model: erroneous source data, or altered loading patterns in vivo. All loads in this model were applied directly and constantly throughout the joint angle range, although muscle loading in vivo involves complex patterns of co‐contraction with constantly varying loads and ratios, which were beyond the scope of this study. Although electromyography (EMG) data exist for some of the relevant muscles,^35^ muscle forces do not always correlate well with EMG activity, being dependent on whether contraction is concentric, isometric, or eccentric.^36^ However, the loads applied were sufficient to cause CTT and to demonstrate the positive effects of hamstring muscle activity on stifle stability. Further analysis of muscle activities at a wider range of joint angles and physical activities is warranted to enable more accurate models in the future.

Both CCLx and MMR caused CTT increases from early in the flexion‐extension movement with increases most pronounced following MMR and in the absence of a hamstring load, with the translation limited in terminal extension due to tension in other joint‐supporting structures. These findings are consistent with previous reports,^23^, ^37^ and suggest that the medial meniscus is a secondary restraint of CTT, as reported in dogs^6^, ^17^, ^23^, ^28^ and humans.^38^ The caudal pole of the medial meniscus may act as a wedge, limiting caudal displacement of the medial femoral condyle:^39^ this role in the CCL‐deficient stifle may expose the medial meniscus to injury due to increased shear forces during weight bearing.^6^, ^28^, ^39^ The integrity of the medial meniscus influences stifle stability after TPLO in vivo, with greater CTT in CCL‐deficient stifles with concurrent hemimeniscectomy.^6^ Both MMR and caudal hemimeniscectomy affect the joint similarly^40^ and performance of either is likely to impact joint stability negatively.

Theoretical studies have stated that if the tibial plateau is inclined perpendicular to the patellar tendon (PTA of 90°), the cranial and caudal shear forces at the articular surfaces will be approach zero during walking.^18^, ^41^ This angle is termed the crossover angle. This crossover angle is achieved at a stifle flexion angle of 90 degrees and represents the point at which cranial tibial thrust and loading of the CCL is converted into caudal tibial thrust and loading of the caudal cruciate ligament.^41^ Our study showed a linear relationship between PTA and joint angle, which is in line with previous studies.^23^, ^41^ Following CCLx and MMR, the PTA curves exhibit periods of limited increase. In the absence of a hamstring load, these periods occur at PTA of 90°‐95° and 85°‐90° for CCLx and MMR, respectively. In contrast, with a hamstring load, these periods occur at 100°‐105° and 94°‐95°. In all cases, these periods are associated with CTT, and thus represent the PTA reaching the crossover angle, with loading shifting from caudal tibial thrust to unconstrained cranial tibial thrust, allowing the tibial tuberosity to move cranially and preventing or slowing the rate of increase in PTA. The varying values of PTA for these periods provide evidence for load‐ and situation‐dependent crossover angles, as has been seen previously in canine and human models.^23^, ^42^ Stifle stability was better at lower caudal joint angles, in agreement with previous studies.^18^, ^41^, ^42^, ^43^ The PTA curve following CBLO is consistent with placement of the joint into relative flexion by rotation of the proximal metaphysis during surgery.

A previous study of TTO demonstrated stability in 5/9 stifles at a caudal joint angle of 125°, slightly better than the 3/7 found here for CBLO with no hamstring load.^23^ This rate of instability in extension is not dissimilar to that seen in vivo with TPLO and TTA,^6^, ^15^, ^44^, ^45^, ^46^ and may reflect the relatively severe quadriceps: gastrocnemius loads employed here, as discussed above. Our TPA values following CBLO are consistent with clinical case series with good reported effects,^7^, ^47^ but even smaller TPA values may not necessarily eliminate CTT.^14^ Mild residual CTT is a stated aim of the CBLO technique, as this should avoid loss of compliance in the cranial joint structures due to cranial subluxation of the femoral condyles, with subsequent intraarticular pathology.^47^, ^48^ Although our model does not simulate weight bearing, and therefore might be more representative of the swing phase, the pattern of stifle stability corresponds well with in vivo findings.

TPLO ex vivo stabilizes the stifle by converting cranial tibial thrust into caudal tibial thrust, resulting in caudal tibial translation and making the caudal cruciate ligament the primary stabilizer of the stifle:^30^ similar results have been seen in vivo, with caudal tibial translation occurring in 10/16 dogs evaluated fluoroscopically.^15^ A similar mode of action might be expected with CBLO. Excessive tibial plateau rotation increases caudal cruciate ligament stresses and could predispose it to fatigue failure or further pathology.^15^, ^30^ Although evaluation of caudal cruciate ligament tension was not among the goals of the present study, only limited caudal tibial translation was seen in the present study with a target TPA of 10°, and it may be that CBLO‐treated stifles will exhibit less stress on the caudal cruciate ligament. This is likely a consequence of the greater postoperative TPA after CBLO compared to TPLO, which typically aims for a TPA of approximately 5°.

Despite this higher TPA, CBLO without hamstring load resulted in normalization of the CTT up to 115° (approximately the normal standing angle), with gradual subluxation thereafter: adding the hamstring load helped to improve stability at these angles but was associated with a mild caudal translation. In contrast, an in silico study of CBLO identified limited caudal tibial translation in extension, which increased in magnitude during a simulated squat to full flexion.^12^ Differences may be due to the model assumptions, including muscle force ratios (initial in silico quadriceps: gastrocnemius ratio of 1:0.61) and lines of action, as well as the choice of bone axes for joint angle calculation.

Interobserver agreement for both spline‐derived variables appeared good, based on the small wsSD values, which define a 67% interval for measurement error. In contrast, the observed ICC values for CCL marker separation distance suggested poor agreement. However, we believe this is artefactual and due to the low underlying variability in this measurement, which makes minor interobserver variation appear more significant than it is.^49^ For PTA, the larger variability in these measurements resulted in ICC values more in line with the wsSD values.

This study has some additional limitations. We used a centroid line of action^50^ for the gastrocnemius muscle, which is caudal to that employed in most previous studies,^28^, ^30^, ^31^, ^32^, ^33^, ^34^ and some divergence occurred at extremes of flexion and extension, which could have impacted joint moments and forces. Additionally, the hamstring group was reduced to a single line of action inserting on the caudal tibial metaphysis for convenience and to avoid disruption of the loading line by the CBLO procedure. This group naturally inserts over a wider region of the metaphysis, and may not be mediolaterally balanced as was the case in this model. The loading line used to control extension may have added additional compressive loading across the joint, with uncertain effects. Stifle stability was only evaluated in the sagittal plane; in vivo, internal rotation is also observed with CCL deficiency and can persist following TPLO.^27^ Internal or external rotation could also affect tibial axis measurement due to bead placement in the cortical bone, and CCL marker separation distance. We believe joint angle errors would be minimal due to the marker beads moving in concert: however, using mean rotational ranges of up to 8.2° reported for intact and CCL deficient stifles,^27^ and assuming the entire rotational effect was restricted to just 1 marker, the angular error would be less than 2.3°. Similarly, CCL marker separation distance would likely be maximally affected by 0.5%, within our experimental error. Separation distances are reported as percentages normalized to 1 stifle joint, and comparison with actual distances should be made with caution. The number of stifles was relatively low and from similarly sized large‐breed dogs: extrapolation of these results to the general canine population may not be appropriate, particularly smaller breeds or those with different limb conformations. The joints used were from healthy animals: periarticular fibrosis and thickening may reduce CTT in vivo and enhance stability after CBLO. We did not assess the effect of CCLx alone with CBLO, and the results presented here therefore represent a worst‐case scenario.

In conclusion, CBLO to a target tibial plateau angle of 10° largely eliminated CTT induced by CCLx and MMR in this ex vivo model. Hamstring loads of 20% quadriceps load improved stifle stability in this model. Stifle stability following CBLO appears to be multifactorial and depends on meniscal integrity, joint angle, and hamstring activation.

## CONFLICT OF INTEREST

The authors declare no conflicts of interest related to this report.

## Supporting information


**Table S1.** Cranial cruciate ligament marker separation changes in intact, cranial cruciate ligament transection (CCLx), medial meniscal release (MMR) and CORA‐based leveling osteotomy (CBLO) situations without additional hamstring load. Statistical output is given by caudal joint angle, with the overall test statistic (Q) and degrees of freedom (df) for Friedmans's ANOVA as well as Holm‐Bonnferoni corrected p‐values for preselected pairwise comparisons with uncorrected p‐values in parentheses. Non‐significant pairwise comparisons are shaded
**Table S2**. Cranial cruciate ligament marker separation changes in intact, cranial cruciate ligament transection (CCLx), medial meniscal release (MMR) and CORA‐based leveling osteotomy (CBLO) situations with additional 29 N hamstring load. Statistical output is given by caudal joint angle, with the overall test statistic (Q) and degrees of freedom (df) for Friedmans's ANOVA as well as Holm‐Bonnferoni corrected p‐values for preselected pairwise comparisons with uncorrected p‐values in parentheses. Non‐significant pairwise comparisons are shaded.
**Table S3**. Comparison of cranial cruciate ligament marker separation distances with 0 N and 29 N hamstring loads for cranial cruciate ligament transection (CCLx), medial meniscal release (MMR) and CORA‐based leveling osteotomy (CBLO). Statistical output is given by caudal joint angle, with the test statistic (Z) and p‐value for Wilcoxon's signed rank test. Median differences and confidence intervals derived using the Hodges‐Lehman estimator are provided. Non‐significant comparisons are shaded
**Table S4**. Patellar ligament (tendon) angle changes in intact, cranial cruciate ligament transection (CCLx), medial meniscal release (MMR) and CORA‐based leveling osteotomy (CBLO) situations without additional hamstring load. Statistical output is given by caudal joint angle, with the overall test statistic (Q) and degrees of freedom (df) for Friedmans's ANOVA as well as Holm‐Bonnferoni corrected p‐values for preselected pairwise comparisons with uncorrected p‐values in parentheses. Non‐significant pairwise comparisons are shaded
**Table S5**. Patellar ligament (tendon) angle changes in intact, cranial cruciate ligament transection (CCLx), medial meniscal release (MMR) and CORA‐based leveling osteotomy (CBLO) situations with additional 29 N hamstring load. Statistical output is given by caudal joint angle, with the overall test statistic (Q) and degrees of freedom (df) for Friedmans's ANOVA as well as Holm‐Bonnferoni corrected p‐values for preselected pairwise comparisons with uncorrected p‐values in parentheses. Non‐significant pairwise comparisons are shaded
**Table S6**. Comparison of patellar ligament (tendon) angles with 0 N and 29 N hamstring loads for cranial cruciate ligament transection (CCLx), medial meniscal release (MMR) and CORA‐based leveling osteotomy (CBLO). Statistical output is given by caudal joint angle, with the test statistic (Z) and p‐value for Wilcoxon's signed rank test. Median differences and confidence intervals derived using the Hodges‐Lehman estimator are provided. Non‐significant comparisons are shaded.
