# Supplementary material for: Effect of center of rotation of angulation‐based leveling osteotomy on ex vivo stifle joint stability following cranial cruciate ligament transection and medial meniscal release with and without a hamstring load
Source: Vet Surg. 2022 Mar 15;51(6):940–51. doi: 10.1111/vsu.13801 (PMC9546295; doi:10.1111/vsu.13801)
Supplement: Supplementary file 1 — Table S1. Cranial cruciate ligament marker separation changes in intact, cranial cruciate ligament transection (CCLx), medial meniscal release (MMR) and CORA‐based leveling osteotomy (CBLO) situations without additional hamstring load. Statistical output is given by caudal joint angle, with the overall test statistic (Q) and degrees of freedom (df) for Friedmans's ANOVA as well as Holm‐Bonnferoni corrected p‐values for preselected pairwise comparisons with uncorrected p‐values in parentheses. Non‐significant pairwise comparisons are shaded Table S2. Cranial cruciate ligament marker separation changes in intact, cranial cruciate ligament transection (CCLx), medial meniscal release (MMR) and CORA‐based leveling osteotomy (CBLO) situations with additional 29 N hamstring load. Statistical output is given by caudal joint angle, with the overall test statistic (Q) and degrees of freedom (df) for Friedmans's ANOVA as well as Holm‐Bonnferoni corrected p‐values for preselected pairwise comparisons with uncorrected p‐values in parentheses. Non‐significant pairwise comparisons are shaded. Table S3. Comparison of cranial cruciate ligament marker separation distances with 0 N and 29 N hamstring loads for cranial cruciate ligament transection (CCLx), medial meniscal release (MMR) and CORA‐based leveling osteotomy (CBLO). Statistical output is given by caudal joint angle, with the test statistic (Z) and p‐value for Wilcoxon's signed rank test. Median differences and confidence intervals derived using the Hodges‐Lehman estimator are provided. Non‐significant comparisons are shaded Table S4. Patellar ligament (tendon) angle changes in intact, cranial cruciate ligament transection (CCLx), medial meniscal release (MMR) and CORA‐based leveling osteotomy (CBLO) situations without additional hamstring load. Statistical output is given by caudal joint angle, with the overall test statistic (Q) and degrees of freedom (df) for Friedmans's ANOVA as well as Holm‐Bonnferoni corrected p‐values [file VSU-51-940-s001.docx]

Supplementary Table 1: Cranial cruciate ligament marker separation changes in intact, cranial cruciate ligament transection (CCLx), medial meniscal release (MMR) and CORA-based leveling osteotomy (CBLO) situations without additional hamstring load. Statistical output is given by caudal joint angle, with the overall test statistic (Q) and degrees of freedom (df) for Friedmans’s ANOVA as well as Holm-Bonnferoni corrected p-values for preselected pairwise comparisons with uncorrected p-values in parentheses. Non-significant pairwise comparisons are shaded.

| Angle | Q (df) | P-value | Intact-CCLx | Intact-MMR | Intact-CBLO | CBLO0-MMR |
| --- | --- | --- | --- | --- | --- | --- |
| 50 | 12.8 (3) | 0.005 | 0.05 (0.02) | 0.02 (0.004) | 0.84 (0.84) | 0.02 (0.007) |
| 55 | 14.8 (3) | 0.002 | 0.3 (0.15) | 0.002 (<0.001) | 0.68 (0.68) | 0.006 (0.002) |
| 60 | 16.2 (3) | 0.001 | 0.05 (0.02) | 0.002 (<0.001) | 0.68 (0.68) | 0.006 (0.002) |
| 65 | 19.3 (3) | <0.001 | 0.13 (0.06) | 0.003 (0.001) | 0.54 (0.54) | <0.001 (<0.001) |
| 70 | 18.9 (3) | <0.001 | 0.08 (0.04) | 0.001 (<0.001) | 0.84 (0.84) | 0.001 (<0.001) |
| 75 | 17.2 (3) | <0.001 | 0.03 (0.01) | 0.006 (0.002) | 0.84 (0.84) | 0.004 (0.001) |
| 80 | 17.2 (3) | 0.001 | 0.03 (0.01) | 0.006 (0.002) | 0.84 (0.84) | 0.004 (0.001) |
| 85 | 17.2 (3) | 0.001 | 0.01 (0.007) | 0.004 (0.001) | 0.84 (0.84) | 0.006 (0.002) |
| 90 | 16.9 (3) | <0.001 | 0.01 (0.004) | 0.008 (0.002) | 0.84 (0.84) | 0.008 (0.004) |
| 95 | 16.9 (3) | 0.001 | 0.01 (0.007) | 0.01 (0.004) | 0.84 (0.84) | 0.008 (0.002) |
| 100 | 14.7 (3) | 0.002 | 0.03 (0.01) | 0.02 (0.004) | 1 (1) | 0.01 (0.004) |
| 105 | 15.2 (3) | 0.002 | 0.05 (0.02) | 0.008 (0.002) | 1 (1) | 0.006 (0.002) |
| 110 | 15.9 (3) | 0.001 | 0.02 (0.007) | 0.002 (<0.001) | 0.41 (0.41) | 0.01 (0.007) |
| 115 | 16.7 (3) | 0.001 | 0.01 (0.004) | 0.001 (<0.001) | 0.21 (0.21) | 0.03 (0.01) |
| 120 | 17.6 (3) | 0.001 | 0.02 (0.007) | <0.001 (<0.001) | 0.21 (0.21) | 0.01 (0.007) |
| 125 | 14.7 (3) | 0.002 | 0.04 (0.01) | 0.001 (<0.001) | 0.1 (0.1) | 0.08 (0.04) |
| 130 | 13.1 (3) | 0.004 | 0.04 (0.01) | 0.002 (<0.001) | 0.13 (0.06) | 0.1 (0.1) |
| 135 | 14.1 (3) | 0.003 | 0.07 (0.02) | 0.001 (<0.001) | 0.13 (0.06) | 0.06 (0.06) |
| 140 | 12.1 (3) | 0.007 | 0.13 (0.06) | 0.008 (0.002) | 0.01 (0.004) | 0.84 (0.84) |

Supplementary Table 2: Cranial cruciate ligament marker separation changes in intact, cranial cruciate ligament transection (CCLx), medial meniscal release (MMR) and CORA-based leveling osteotomy (CBLO) situations with additional 29 N hamstring load. Statistical output is given by caudal joint angle, with the overall test statistic (Q) and degrees of freedom (df) for Friedmans’s ANOVA as well as Holm-Bonnferoni corrected p-values for preselected pairwise comparisons with uncorrected p-values in parentheses. Non-significant pairwise comparisons are shaded.

| Angle | Q (df) | P-value | Intact-CCLx | Intact-MMR | Intact-CBLO | CBLOB3-MMR |
| --- | --- | --- | --- | --- | --- | --- |
| 50 | 0.943 (3) | 0.82 | - | - | - | - |
| 55 | 1.8 (3) | 0.62 | - | - | - | - |
| 60 | 2.49 (3) | 0.48 | - | - | - | - |
| 65 | 2.49 (3) | 0.48 | - | - | - | - |
| 70 | 3.51 (3) | 0.32 | - | - | - | - |
| 75 | 3.86 (3) | 0.28 | - | - | - | - |
| 80 | 3.17 (3) | 0.37 | - | - | - | - |
| 85 | 4.2 (3) | 0.24 | - | - | - | - |
| 90 | 7.29 (3) | 0.06 | - | - | - | - |
| 95 | 6.6 (3) | 0.09 | - | - | - | - |
| 100 | 10.4 (3) | 0.02 | 0.54 (0.54) | 0.82 (0.41) | 0.07 (0.02) | 0.008 (0.002) |
| 105 | 14.7 (3) | 0.002 | 0.41 (0.41) | 0.2 (0.1) | 0.12 (0.04) | 0.001 (<0.001) |
| 110 | 13.1 (3) | 0.004 | 0.54 (0.54) | 0.2 (0.1) | 0.19 (0.06) | 0.002 (<0.001) |
| 115 | 17.9 (3) | <0.001 | 0.41 (0.41) | 0.07 (0.02) | 0.13 (0.06) | <0.001 (<0.001) |
| 120 | 17.6 (3) | <0.001 | 0.3 (0.15) | 0.02 (0.007) | 0.21 (0.21) | <0.001 (<0.001) |
| 125 | 18.3 (3) | <0.001 | 0.08 (0.04) | 0.006 (0.002) | 0.54 (0.54) | 0.001 (<0.001) |
| 130 | 17.9 (3) | <0.001 | 0.05 (0.02) | 0.003 (0.001) | 0.84 (0.84) | 0.002 (<0.001) |
| 135 | 16.2 (3) | 0.001 | 0.13 (0.06) | 0.006 (0.002) | 0.68 (0.68) | 0.002 (<0.001) |
| 140 | 13.5 (3) | 0.004 | 0.13 (0.06) | 0.01 (0.004) | 0.84 (0.84) | 0.008 (0.002) |

Supplementary Table 3: Comparison of cranial cruciate ligament marker separation distances with 0 N and 29 N hamstring loads for cranial cruciate ligament transection (CCLx), medial meniscal release (MMR) and CORA-based leveling osteotomy (CBLO). Statistical output is given by caudal joint angle, with the test statistic (Z) and p-value for Wilcoxon’s signed rank test. Median differences and confidence intervals derived using the Hodges-Lehman estimator are provided. Non-significant comparisons are shaded.

|  | CCLx | | | MMR | | | CBLO | | |
| --- | --- | --- | --- | --- | --- | --- | --- | --- | --- |
| Angle | Z | P-value | Median difference | Z | P-value | Median difference | Z | P-value | Median difference |
| 50 | -2.4 | 0.018 | -0.087  (-0.18; -0.043) | -2.4 | 0.018 | -0.24  (-0.41; -0.045) | -0.85 | 0.40 | -0.017  (-0.055; 0.03) |
| 55 | -2.4 | 0.018 | -0.1  (-0.2; -0.031) | -2.4 | 0.018 | -0.29  (-0.54; -0.059) | -1.5 | 0.13 | -0.028  (-0.054; 0.01) |
| 60 | -2.4 | 0.018 | -0.12  (-0.22; -0.046) | -2.4 | 0.018 | -0.31  (-0.57; -0.12) | -1.5 | 0.13 | -0.022  (-0.056; 0.017) |
| 65 | -2.4 | 0.018 | -0.14  (-0.25; -0.057) | -2.4 | 0.018 | -0.34  (-0.64; -0.15) | -1.5 | 0.13 | -0.022  (-0.054; 0.0082) |
| 70 | -2.4 | 0.018 | -0.17  (-0.29; -0.1) | -2.4 | 0.018 | -0.36  (-0.68; -0.17) | -1.9 | 0.06 | -0.029  (-0.062; 0.0013) |
| 75 | -2.4 | 0.018 | -0.2  (-0.33; -0.12) | -2.4 | 0.018 | -0.38  (-0.68; -0.18) | -1.9 | 0.06 | -0.021  (-0.07; 0.0094) |
| 80 | -2.4 | 0.018 | -0.24  (-0.37; -0.15) | -2.4 | 0.018 | -0.41  (-0.69; -0.19) | -2 | 0.04 | -0.026  (-0.065; -0.0014) |
| 85 | -2.4 | 0.018 | -0.29  (-0.42; -0.18) | -2.4 | 0.018 | -0.41  (-0.69; -0.18) | -2.2 | 0.028 | -0.025  (-0.045; -0.0078) |
| 90 | -2.4 | 0.018 | -0.34  (-0.45; -0.2) | -2.4 | 0.018 | -0.39  (-0.7; -0.18) | -2.4 | 0.018 | -0.032  (-0.082; -0.0042) |
| 95 | -2.4 | 0.018 | -0.4  (-0.52; -0.21) | -2.4 | 0.018 | -0.4  (-0.63; -0.17) | -2.2 | 0.028 | -0.063  (-0.21; -0.01) |
| 100 | -2.4 | 0.018 | -0.39  (-0.54; -0.22) | -2.4 | 0.018 | -0.28  (-0.46; -0.17) | -2 | 0.04 | -0.083  (-0.35; -0.0035) |
| 105 | -2.4 | 0.018 | -0.36  (-0.5; -0.21) | -2.4 | 0.018 | -0.2  (-0.32; -0.11) | -2 | 0.04 | -0.091  (-0.39; -0.0074) |
| 110 | -2.4 | 0.018 | -0.36  (-0.48; -0.2) | -2.4 | 0.018 | -0.16  (-0.26; -0.058) | -2.2 | 0.028 | -0.11  (-0.39; -0.025) |
| 115 | -2.4 | 0.018 | -0.29  (-0.42; -0.15) | -2.4 | 0.018 | -0.14  (-0.24; -0.039) | -2.4 | 0.018 | -0.12  (-0.38; -0.048) |
| 120 | -2.4 | 0.018 | -0.24  (-0.41; -0.11) | -2.4 | 0.018 | -0.11  (-0.24; -0.026) | -2.2 | 0.028 | -0.21  (-0.45; -0.049) |
| 125 | -2.4 | 0.018 | -0.19  (-0.36; -0.078) | -2.4 | 0.018 | -0.1  (-0.18; -0.034) | -2.2 | 0.028 | -0.27  (-0.48; -0.069) |
| 130 | -2.4 | 0.018 | -0.2  (-0.38; -0.049) | -2.4 | 0.018 | -0.078  (-0.14; -0.034) | -2.2 | 0.028 | -0.26  (-0.46; -0.072) |
| 135 | -2.4 | 0.018 | -0.18  (-0.37; -0.011) | -2.4 | 0.018 | -0.072  (-0.13; -0.019) | -2.2 | 0.028 | -0.31  (-0.44; -0.056) |
| 140 | -1.5 | 0.13 | -0.14  (-0.34; 0.044) | -2 | 0.04 | -0.056  (-0.12; -0.01) | -2.4 | 0.018 | -0.28  (-0.44; -0.048) |

Supplementary Table 4: Patellar ligament (tendon) angle changes in intact, cranial cruciate ligament transection (CCLx), medial meniscal release (MMR) and CORA-based leveling osteotomy (CBLO) situations without additional hamstring load. Statistical output is given by caudal joint angle, with the overall test statistic (Q) and degrees of freedom (df) for Friedmans’s ANOVA as well as Holm-Bonnferoni corrected p-values for preselected pairwise comparisons with uncorrected p-values in parentheses. Non-significant pairwise comparisons are shaded.

| Angle | Q (df) | P-value | Intact-CCLx | Intact-MMR | Intact-CBLO | CBLO-MMR |
| --- | --- | --- | --- | --- | --- | --- |
| 50 | 16.2 (3) | 0.001 | 0.12 (0.06) | 0.006 (0.002) | <0.001 (<0.001) | 0.53 (0.53) |
| 55 | 13.8 (3) | 0.003 | 0.05 (0.02) | 0.006 (0.002) | 0.004 (<0.001) | 0.84 (0.84) |
| 60 | 14.5 (3) | 0.002 | 0.29 (0.15) | 0.006 (0.002) | 0.004 (<0.001) | 0.84 (0.84) |
| 65 | 16 (3) | 0.001 | 0.43 (0.21) | 0.004 (<0.001) | 0.003 (<0.001) | 1.0 (1.0) |
| 70 | 16 (3) | 0.001 | 0.43 (0.21) | 0.004 (<0.001) | 0.003 (<0.001) | 1.0 (1.0) |
| 75 | 15.9 (3) | 0.001 | 0.12 (0.06) | 0.002 (<0.001) | 0.003 (<0.001) | 0.84 (0.84) |
| 80 | 13.1 (3) | 0.004 | 0.03 (0.01) | 0.008 (0.002) | 0.006 (0.002) | 1.0 (1.0) |
| 85 | 11.2 (3) | 0.011 | 0.08 (0.04) | 0.02 (0.004) | 0.01 (0.004) | 1.0 (1.0) |
| 90 | 13.3 (3) | 0.004 | 0.03 (0.01) | 0.004 (<0.001) | 0.01 (0.004) | 0.68 (0.68) |
| 95 | 13.8 (3) | 0.003 | 0.03 (0.01) | 0.002 (<0.001) | 0.02 (0.007) | 0.41 (0.41) |
| 100 | 13.1 (3) | 0.004 | 0.02 (0.007) | 0.004 (<0.001) | 0.01 (0.007) | 0.53 (0.53) |
| 105 | 13.8 (3) | 0.003 | 0.02 (0.007) | 0.002 (<0.001) | 0.03 (0.01) | 0.30 (0.30) |
| 110 | 14.7 (3) | 0.002 | 0.04 (0.01) | <0.001 (<0.001) | 0.03 (0.01) | 0.21 (0.21) |
| 115 | 13.8 (3) | 0.003 | 0.03 (0.01) | 0.002 (<0.001) | 0.02 (0.007) | 0.41 (0.41) |
| 120 | 13.3 (3) | 0.004 | 0.03 (0.01) | 0.004 (<0.001) | 0.01 (0.004) | 0.68 (0.68) |
| 125 | 14.7 (3) | 0.002 | 0.04 (0.01) | <0.001 (<0.001) | 0.03 (0.01) | 0.21 (0.21) |
| 130 | 14.8 (3) | 0.002 | 0.08 (0.04) | 0.002 (<0.001) | 0.006 (0.002) | 0.68 (0.68) |
| 135 | 14.7 (3) | 0.002 | 0.08 (0.04) | 0.004 (<0.001) | 0.003 (<0.001) | 1.0 (1.0) |
| 140 | 14.7 (3) | 0.002 | 0.08 (0.04) | 0.004 (<0.001) | 0.003 (<0.001) | 1.0 (1.0) |

Supplementary Table 5: Patellar ligament (tendon) angle changes in intact, cranial cruciate ligament transection (CCLx), medial meniscal release (MMR) and CORA-based leveling osteotomy (CBLO) situations with additional 29 N hamstring load. Statistical output is given by caudal joint angle, with the overall test statistic (Q) and degrees of freedom (df) for Friedmans’s ANOVA as well as Holm-Bonnferoni corrected p-values for preselected pairwise comparisons with uncorrected p-values in parentheses. Non-significant pairwise comparisons are shaded.

| Angle | Q (df) | P-value | Intact-CCLx | Intact-MMR | Intact-CBLO | CBLO-MMR |
| --- | --- | --- | --- | --- | --- | --- |
| 50 | 13.3 (3) | 0.004 | 0.82 (0.41) | 0.68 (0.68) | 0.04 (0.01) | 0.02 (0.004) |
| 55 | 13.1 (3) | 0.004 | 1.0 (0.53) | 0.53 (0.53) | 0.04 (0.01) | 0.008 (0.002) |
| 60 | 13.3 (3) | 0.004 | 0.82 (0.41) | 0.68 (0.68) | 0.04 (0.01) | 0.02 (0.004) |
| 65 | 14.7 (3) | 0.002 | 1.0 (1.0) | 0.43 (0.21) | 0.04 (0.01) | <0.001 (<0.001) |
| 70 | 14.8 (3) | 0.002 | 0.68 (0.68) | 0.29 (0.15) | 0.07 (0.02) | <0.001 (<0.001) |
| 75 | 14.1 (3) | 0.003 | 1.0 (0.53) | 0.53 (0.53) | 0.01 (0.004) | 0.002 (<0.001) |
| 80 | 13.3 (3) | 0.004 | 1.0 (0.68) | 0.68 (0.68) | 0.01 (0.004) | 0.004 (<0.001) |
| 85 | 13.1 (3) | 0.004 | 1.0 (0.53) | 0.53 (0.53) | 0.04 (0.01) | 0.008 (0.002) |
| 90 | 12.6 (3) | 0.006 | 1.0 (1.0) | 1.0 (1.0) | 0.02 (0.004) | 0.01 (0.004) |
| 95 | 16.2 (3) | 0.001 | 0.12 (0.06) | 0.21 (0.21) | <0.001 (<0.001) | 0.02 (0.007) |
| 100 | 14.7 (3) | 0.002 | 0.43 (0.21) | 0.21 (0.21) | <0.001 (<0.001) | 0.04 (0.01) |
| 105 | 12.8 (3) | 0.005 | 1.0 (0.53) | 0.02 (0.007) | 0.02 (0.004) | 0.84 (0.84) |
| 110 | 11.2 (3) | 0.01 | 0.82 (0.41) | 0.02 (0.004) | 0.04 (0.01) | 0.68 (0.68) |
| 115 | 12.1 (3) | 0.007 | 0.43 (0.21) | 0.008 (0.002) | 0.02 (0.007) | 0.68 (0.68) |
| 120 | 12.4 (3) | 0.006 | 0.12 (0.06) | 0.004 (<0.001) | 0.02 (0.007) | 0.53 (0.53) |
| 125 | 16.2 (3) | 0.001 | 0.08 (0.04) | <0.001 (<0.001) | 0.02 (0.007) | 0.21 (0.21) |
| 130 | 16.2 (3) | 0.001 | 0.08 (0.04) | <0.001 (<0.001) | 0.02 (0.007) | 0.21 (0.21) |
| 135 | 16.2 (3) | 0.001 | 0.12 (0.06) | <0.001 (<0.001) | 0.006 (0.002) | 0.53 (0.53) |
| 140 | 14.8 (3) | 0.002 | 0.29 (0.15) | 0.01 (0.004) | 0.002 (<0.001) | 0.53 (0.53) |

Supplementary Table 6: Comparison of patellar ligament (tendon) angles with 0 N and 29 N hamstring loads for cranial cruciate ligament transection (CCLx), medial meniscal release (MMR) and CORA-based leveling osteotomy (CBLO). Statistical output is given by caudal joint angle, with the test statistic (Z) and p-value for Wilcoxon’s signed rank test. Median differences and confidence intervals derived using the Hodges-Lehman estimator are provided. Non-significant comparisons are shaded.

|  | CCLx | | | MMR | | | CBLO | | |
| --- | --- | --- | --- | --- | --- | --- | --- | --- | --- |
| Angle | Z | P-value | Median difference | Z | P-value | Median difference | Z | P-value | Median difference |
| 50 | 2.4 | 0.02 | 2.7 (1; 5.9) | 2.4 | 0.02 | 6.3 (2.9; 12) | -0.85 | 0.40 | -1.5 (-6.4; 3.1) |
| 55 | 2.4 | 0.02 | 3.8 (1.4; 7.1) | 2.4 | 0.02 | 8.3 (3; 13) | -1.2 | 0.24 | -1.4 (-4.1; 1.4) |
| 60 | 1.9 | 0.06 | 2.7 (-0.78; 6.6) | 2.4 | 0.02 | 8.9 (3.1; 14) | -0.68 | 0.50 | -0.44 (-2.9; 0.96) |
| 65 | 2 | 0.04 | 2.2 (0.034; 7.6) | 2.4 | 0.02 | 10 (3.8; 17) | 0 | 1.0 | -0.068 (-3; 1.7) |
| 70 | 1.9 | 0.06 | 2.9 (-0.13; 7.6) | 2.4 | 0.02 | 12 (5.9; 18) | -1.2 | 0.24 | -1.1 (-4.4; 1.3) |
| 75 | 2.4 | 0.02 | 4.5 (0.8; 9.2) | 2.4 | 0.02 | 13 (5.3; 19) | -1.5 | 0.13 | -1.7 (-4.4; 0.75) |
| 80 | 2.4 | 0.02 | 6.3 (2.4; 11) | 2.4 | 0.02 | 12 (5.1; 20) | -0.68 | 0.50 | -0.37 (-3.6; 0.59) |
| 85 | 2.4 | 0.02 | 6.7 (3.1; 12) | 2.4 | 0.02 | 13 (5.6; 20) | -0.34 | 0.74 | -0.29 (-2.5; 0.99) |
| 90 | 2.4 | 0.02 | 7.0 (4.2; 12) | 2.4 | 0.02 | 13 (5.6; 20) | -1.7 | 0.09 | -1.5 (-2.8; 0.69) |
| 95 | 2.4 | 0.02 | 7.3 (4.4; 14) | 2.4 | 0.02 | 12 (5.6; 16) | -1.2 | 0.24 | -1.8 (-2.9; 2.1) |
| 100 | 2.4 | 0.02 | 7.6 (3.2; 15) | 2.4 | 0.02 | 9.3 (5.3; 13) | -0.34 | 0.74 | -0.32 (-3.7; 5.4) |
| 105 | 2.4 | 0.02 | 6.6 (3.6; 14) | 2.4 | 0.02 | 6.1 (3.2; 10) | 1 | 0.31 | 1.4 (-1.7; 6.6) |
| 110 | 2.4 | 0.02 | 6.9 (4; 13) | 2.4 | 0.02 | 4.8 (1.5; 8.8) | 1.4 | 0.18 | 1.9 (-1.2; 6.7) |
| 115 | 2.4 | 0.02 | 7.3 (3.4; 13) | 2.2 | 0.03 | 3.8 (0.66; 8.5) | 1.7 | 0.09 | 3.3 (-0.41; 8.4) |
| 120 | 2.4 | 0.02 | 6.0 (2.2; 9.5) | 2.2 | 0.03 | 2.9 (0.6; 7.7) | 2 | 0.04 | 5.4 (0.048; 10) |
| 125 | 2.4 | 0.02 | 4.9 (1.8; 7.6) | 1.9 | 0.06 | 3.9 (-0.16; 9.3) | 2.4 | 0.02 | 6.0 (0.54; 12) |
| 130 | 2.2 | 0.03 | 4.8 (0.43; 8.8) | 1.4 | 0.18 | 3.0 (-0.96; 7.7) | 2.2 | 0.03 | 6.4 (1.4; 12) |
| 135 | 2 | 0.04 | 4.5 (0.63; 9.2) | 1.5 | 0.13 | 2.3 (-0.68; 7.5) | 2.4 | 0.02 | 6.4 (0.57; 11) |
| 140 | 0.68 | 0.5 | 2.5 (-5.6; 9.1) | 2 | 0.04 | 2.9 (0.19; 6.6) | 2.2 | 0.03 | 6.4 (1.2; 11) |
